# A dual model of normal vs isogenic Nrf2-depleted murine epithelial cells to explore oxidative stress involvement

**DOI:** 10.1038/s41598-024-60938-2

**Published:** 2024-05-13

**Authors:** Jacques Dupuy, Edwin Fouché, Céline Noirot, Pierre Martin, Charline Buisson, Françoise Guéraud, Fabrice Pierre, Cécile Héliès-Toussaint

**Affiliations:** 1 National Research Institute for Agriculture and Environment (INRAE), Toxalim (Research Centre in Food Toxicology), INRAE, ENVT, INP-Purpan, UPS, Université de Toulouse, 180 Chemin de Tournefeuille, BP93173, 31027 Toulouse Cedex 3, France; 2 National Research Institute for Agriculture and Environment (INRAE), Université Fédérale de Toulouse, INRAE, BioinfOmics, GenoToul Bioinformatics Facility, 31326 Castanet-Tolosan, France

**Keywords:** Nrf2, Oxidative stress, Dual-cell model, HNE, Cell signalling, Metabolic pathways

## Abstract

Cancer-derived cell lines are useful tools for studying cellular metabolism and xenobiotic toxicity, but they are not suitable for modeling the biological effects of food contaminants or natural biomolecules on healthy colonic epithelial cells in a normal genetic context. The toxicological properties of such compounds may rely on their oxidative properties. Therefore, it appears to be necessary to develop a dual-cell model in a normal genetic context that allows to define the importance of oxidative stress in the observed toxicity. Given that the transcription factor nuclear factor erythroid 2-related factor 2 (Nrf2) is considered to be the master regulator of antioxidant defenses, our aim was to develop a cellular model comparing normal and Nrf2-depleted isogenic cells to qualify oxidative stress–related toxicity. We generated these cells by using the CRISPR/Cas9 technique. Whole-genome sequencing enabled us to confirm that our cell lines were free of cancer-related mutations. We used 4-hydroxy-2-nonenal (HNE), a lipid peroxidation product closely related to oxidative stress, as a model molecule. Here we report significant differences between the two cell lines in glutathione levels, gene regulation, and cell viability after HNE treatment. The results support the ability of our dual-cell model to study the role of oxidative stress in xenobiotic toxicity.

## Introduction

Intestinal cells are confronted to a large number of xenobiotics, including environmental and dietary contaminants, neo-formed products of the diet, and biomolecules that are naturally produced during digestion. These compounds can exhibit cytotoxic and genotoxic properties in the colon, mainly due to the generation of reactive oxygen species (ROS) and lipid peroxidation products. Under normal conditions, ROS are essential for normal life as they are responsible for maintaining cellular redox homeostasis^1^ and have been considered major signaling molecules^2,3^. However, high ROS concentrations lead to an imbalance between ROS production and the cellular antioxidant systems, resulting in oxidative stress. When cellular repair capacities are exceeded, excessive levels of ROS, electrophiles, or prooxidant molecules can lead to protein or DNA adducts that could contribute to increased oncogenic risk^4^. Given that the transcription factor nuclear factor erythroid 2-related factor 2 (Nrf2) is a master regulator of ROS metabolism and the antioxidant defense, and that its induction leads to protection against oxidative damage^5–7^, we thought it would be of great interest to assess the relative weight of oxidative stress in xenobiotic management.

Due to their availability and ease of use, cell lines represent a tool of choice in xenobiotic toxicity studies. Although cell lines are very useful for modeling cancer promotion and progression, their carcinogenic phenotype and genotype mean that they are poorly suited for studying the biological effects of food contaminants on healthy colonic mucosa. Therefore, we believed it would be appropriate to develop a cellular model with a controlled and normal genetic background to avoid mutations due to carcinogenic transformation, which could distort mechanistic conclusions on contaminant toxicity. We used a normal murine cell model to assess contaminant toxicity in a normal, controlled genetic context. We developed this cellular model from conditionally immortalized colonic epithelial cell lines derived from C57BL/6J mice^8^, and generated an isogenic Nrf2-knockdown cell line by using the CRISPR/Cas9 technique. The normal genetic background of these cell lines was confirmed by whole-genome sequencing (WGS). After generating this dual-cell model, we used 4-hydroxy-2-nonenal (HNE), a molecule formed in the intestine and colonic lumen in a pro-peroxidant context—for example, after a diet high in heme iron and low in antioxidants^9^—as a model of reactive product of lipid peroxidation that induces oxidative stress. The effects of HNE on normal colon cells and their isogenic counterparts lacking Nrf2 enabled us to identify the burden of oxidative stress in cellular responses to xenobiotic attack in our murine epithelial cells.

## Results

### Identification of a normal colonic epithelial cell line

The murine colonic epithelial cell line used in these experiments (named Co) is derived from the Apc^+/+^ clones described by Forest et al.^8^. In these experiments, we used WGS to determine the non-precancerous and non-cancerous status of the Co cell line, with a particular focus on pro-oncogenes, tumor suppressor genes, and high-impact mutations (see Table 1a for sample information). More specifically, we studied mutations in a panel of genes from the main pathways linked to colorectal cancer: the WNT and NOTCH pathways, the c-myc gene, the PI3K and P53 genes and other cancer-related genes, Nrf2 and the associated Nrf1 and Nrf3 genes, the Kelch-like ECH-associated protein 1 (Keap1) gene, and genes involved in cell cycle regulation. Only mutations with a “high” impact, defined by McLaren et al.^10^ as “the variant is assumed to have a disruptive impact in the protein, probably causing protein truncation, loss of function or triggering nonsense-mediated decay,” and a moderate impact, “a non-disruptive variant that might change protein effectiveness,” were considered. With the exception of Notch1 and Cdk6, there are no “high” mutations in the Co cell line (Table 1b; Table S4), confirming its non-cancerous status.

**Table 1:** Sequence comparison analyses.

(a) Raw data								
Sample name	Raw reads	% Duplicates	Reads aligned	≥ 10X	Mean cov	Vars	SNP	Indel
Co	481,788,378	29.6%	479,112,014	45.03	17.39	1,354,919	993,532	362,827

(b) Variant detection in a panel of genes from the main colorectal cancer–related pathways								
Pathways	Genes tested	Transcript length	Average depth (*Mosdeth*)	Feature count on exons	Gene filter	Moderate	High	
WNT	APC	15,753	14.27	1710	9			
	CTNNb1/Beta catenin	5099	50.23	736	5	1		
	GSK3 beta	9821	11.49	3248	10			
	Axin 1	3915	28.44	1662	14			
	Axin 2	5506	36.50	2768	7			
NOTCH	NOTCH 1	14,232	38.73	7990	12	1	1	
	NOTCH 2	10,777	24.76	4501	20			
	NOTCH 3	8044	64.99	6399	10	1		
	NOTCH 4	8080	35.11	5541	43			
myc	c-myc	2767	35.26	1155	1			
TGF beta	SMAD 2	2037	11.51	104	4			
	SMAD 3	5542	64.97	883	11			
	SMAD 4	3741	21.34	631	12	1		
	BMP4	2159	75.70	1411	3			
Hippo	Yap1	9257	13.06	1978	38			
PI3K	Akt1	3948	14.27	2955				
	BRCA1	8533	13.15	786	9			
P53	TRP 53	4056	29.77	1069	6			
Cell cycle	Cdk2	2432	71.45	1385	2			
	Cdk4	3249	13.52	1978	1			
	Cdk6	3151	33.03	1132	2678	12	3	
	E2f1	3150	87.34	2670	13			
	E2f3	4794	38.12	790	8			
	cdkn2a	1172	48.88	893	9			
Others	B raf	13,863	12.48	1755	20			
	K ras	5347	25.54	1035	1			
	PTEN	10,632	16.14	1060	7			
	COX-2	4580	12.54	672				
	Fas	1801	14.84	169	10			
	Bcl2	9129	7.14	2195	13			
NRF2	Nrf1	5350	83.79	3783	2	1		
	Nrf2	3796	20.67	702	4			
	Nrf3	2973	43.92	1663	8			
	Keap1	4664	24.99	2978	7	1		

### Creation of a Nrf2-knockdown clonal cell line using a CRISPR-directed gene editing approach

#### Clone selection

**Figure 1:**
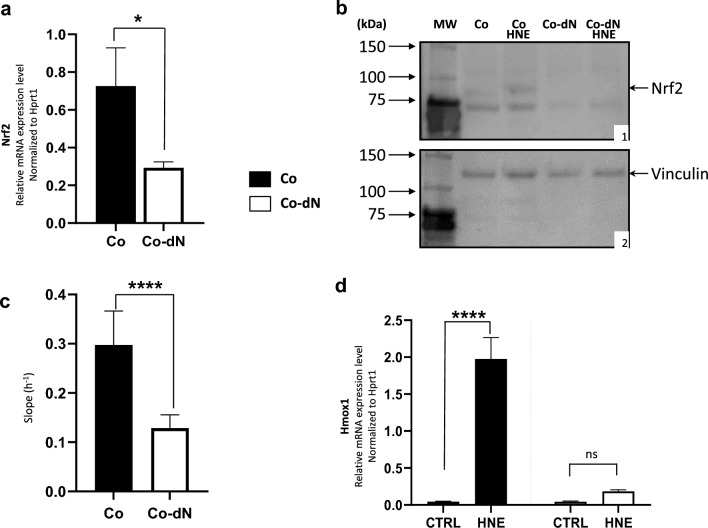
Generation and characterization of a normal mouse colonic epithelial cell line in which the Nrf2 gene is disabled. (**a**) Relative mRNA expression level of *Nrf2*, normalized to the *Hprt1* gene in Co and Co-dN cells. (**b**) Western blot analysis of Nrf2 protein expression in Co and Co-dN cells without or with HNE treatment (40 µM for 6 h); vinculin (124 kDa) was used as an internal control. (**b1**) Molecular weight marker; Nrf2 protein in Co (+/− HNE) and Co-dN (+/− HNE) cell extracts. (**b2**) Molecular weight marker; vinculin protein in Co (+/− HNE) and Co-dN (+/− HNE) cell extracts. (**c**). Reported slope before HNE treatment of both cell types based on xCELLigence data. (**d**) Relative mRNA expression level of *Hmox1*, a target gene of the transcription factor Nrf2. The detoxification pathway was induced by treating cells with HNE (40 µM for 6 h). Gene expression was normalized to the control gene *Hprt1*. For each figure, black boxes are Co cells, open boxes are Co-dN cells, and error bars indicate the mean ± standard error of the mean (n = 3 for **a** and **d**, and n = 5 for **c**). Student t-test (**a**, **c**) or two-way ANOVA followed by Tukey’s multiple comparison test (**d**) were used for statistical analysis (ns = non-significant, *P < 0.05, ****P < 0.0001).

We used the CRISPR/Cas9 technique to disable the Nrf2 gene in the Co cell line, rendering it incapable of producing a functional protein. We detected Nrf2 gene mutations with polymerase chain reaction (PCR), using primers specific to each guide RNA (Supplementary Fig. S1a). After prescreening to identify mismatches, we selected four clones and analyzed Nrf2 messenger RNA (mRNA) expression with reverse transcription–quantitative polymerase chain reaction (RT-qPCR) to select the clones with a substantial level of Nrf2 invalidation. We used hypoxanthine phosphoribosyl transferase 1 (*Hprt1*) as a control gene. We selected one clone, named Co-dN (for colon cell depleted for Nrf2) in which the Nrf2 mRNA level was 60% lower than in the parental Co cell line (Fig. 1a).

#### NRF2 protein expression

We assessed Nrf2 protein expression with a western blot of total protein extracts from the Co and Co-dN cell lines, treated or not with HNE to induce Nrf2 expression (Fig. 1b; S2a). Vinculin served as an internal control (Fig. 1b; S2b). There were two bands in the Co cell line (around 75 and 90 kDa). The 90 kDa band was more intense in HNE-treated Co cells (about 200%, Supplementary Table S2). There was no band in Co-dN cells, without or with HNE treatment. The 90 kDa band is specific to the Nrf2 protein, as reported by Kopacz et al.^11^, while the 75 kDa band corresponds to a non-specific band. Taken together, we detected the Nrf2 protein in Co cells, induced after HNE treatment, a finding that correlates with heme oxygenase 1 (*Hmox1*) mRNA expression (Fig. 1d). On the other hand, there was no Nrf2-specific band in the Co-dN cells even after HNE stimulation, confirming that the active form of Nrf2 had been genetically disabled.

#### Cellular proliferation

The xCELLigence technique enables continuous monitoring of morphological changes in adherent cells^12^. We used this technique to analyze cell proliferation in real time. The initial mean slope of the proliferation curve was 0.297 ± 0.014 h^−1^ for Co cells and 0.128 ± 0.006 h^−1^ for Co-dN cells. Thus, there was a significant (P < 0.0001) 57% reduction in the proliferation rate of the Nrf2-depleted Co-dN cell line (Fig. 1c). The difference between the two groups was analyzed with Student’s *t*-test.

#### Nrf2 target gene induction

We analyzed the modulation of the Nrf2 pathway via a target gene: *Hmox1*. We treated Co and Co-dN cells with HNE (40 μM for 6 h) to activate the Nrf2-mediated HNE detoxification pathway. *Hmox1* was strongly induced by HNE in Co cells (43-fold, P < 0.0001), whereas induction was 10 times weaker in Co-dN cells (4.3-fold, non-significant; Fig. 1d). The differences were assessed with two-way analysis of variance (ANOVA) followed by Tukey’s multiple comparison test.

#### Sequence analysis

**Figure 2:**
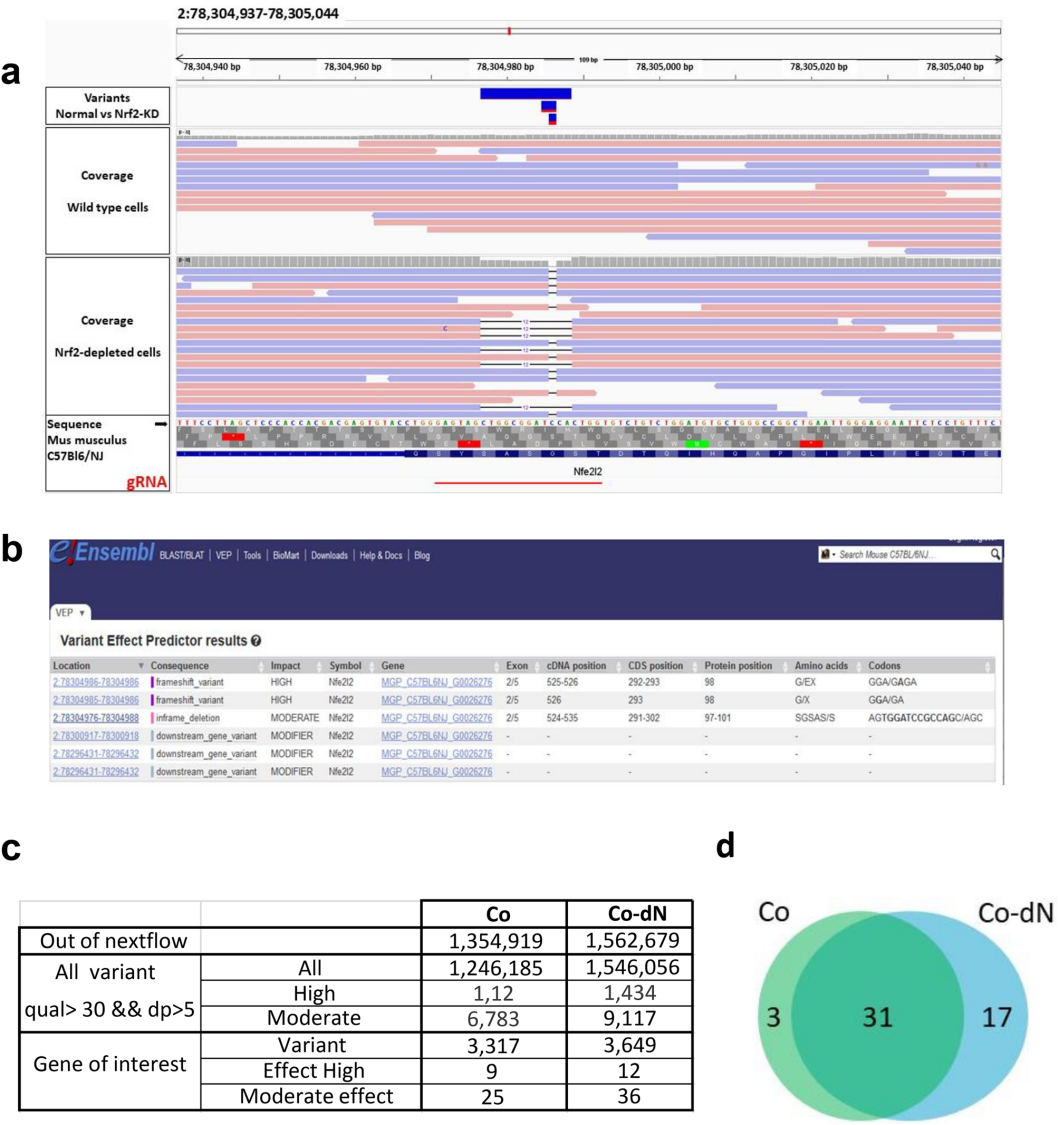
Identification of the deletion of the Nrf2 gene and exploration of variants. (**a**) Identification of insertion/deletion in the Nrf2 gene, visualized using the IGV software and gRNA localization. (**b**) Variants predictor results (extracted from e!Ensembl) showing the “high”-impact mutation in exon 2 of the *Nfe2I2* cDNA. (**c**) The number of variants per gene of interest and per effect. (**d**) Venn diagram representing common variants with high and moderate effects between the two cell lines.

We performed WGS on the Co-dN cell line. The IGV software allowed us to visualize insertion/deletion (indel) in the Nrf2 gene. There is a deletion (Fig. 2a) in exon 2 (Fig. 2b). The search for variants between the initial mouse genotype and the exomes of the two cell lines revealed few differences (Fig. 2c, d; Supplementary Table S3). The Venn diagram (Fig. 2d) shows the total number of variants, which are referenced in supplementary Table S4. A large number of variants: 12 and 22 variants for Co and Co-dN respectively are associated to a predicted gene 16505 (Gm 16505, MGP_C57BL6NJ_G0029685) without any identified function (Table S4). In the Co cell line, 3 genes presented mutations, Notch 1 and 3 and Smad4 but only Notch1 was associated with high impact mutation. In the Co-dN cell line, the *Cdk6* mutation already identified in Co cells is still detectable, confirming that the two cell lines are isogenic. The Notch4 and Trp53 mutations were considered as moderate. Only Nrf2 was associated with high impact mutation (protein modification). There are no other “high” mutations in Co-dN cells, indicating that in our experiment, the CRISPR/Cas9 technique did not induce off-target effects.

### Cellular response to HNE

#### Cellular detoxification

**Figure 3:**
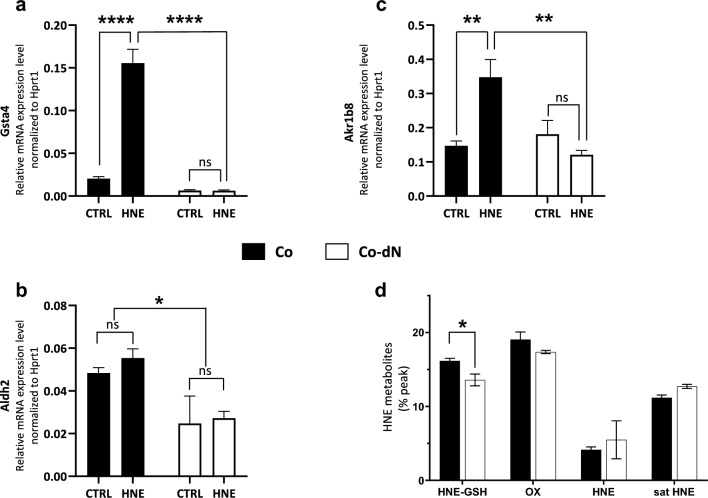
Exploration of the cellular detoxification capacities of the two cell lines. (**a**)–(**c**) Relative expression of Nrf2 target genes (*Gsta4*, *Akr1b8*, and *Aldh2*) in Co and Co-dN cells. The detoxification pathway was induced by treating cells with HNE (40 µM for 6 h). Gene expression was normalized to *Hprt1* expression. (**d)** Identification of HNE metabolites in both cell lines. [^3^H]-HNE (40 µM for 30 min) was used as a radioactive tracer. The cell supernatant was analyzed by radio-HPLC. The results are expressed as the percentage of HPLC peaks. Black boxes correspond to Co cells, open boxes to Co-dN cells, and error bars indicate the mean ± standard error of the mean from three independent experiments. Two-way ANOVA followed by Tukey’s multiple comparison test was used for statistical analysis (ns = non-significant, *P < 0.05, **P < 0.01, ****P < 0.0001).

To investigate the role of Nrf2 in the cellular response to toxic lipid oxidation products, we used RT-qPCR to analyze the expression of three Nrf2-target genes involved in HNE detoxification: glutathione-*S*-transferase A4 (*Gsta4*), aldo–keto reductase 1b8 (*Akr1b8*), and aldehyde dehydrogenase 2 (*Aldh2*)^13^ in both cell lines. We used HNE treatment (40 µM for 6 h) to induce its own detoxification pathway. The results are presented in Fig. 3a–c. Under control conditions, there was no difference between the cell lines in *Gsta4* and *Akr1b8* expression, but Nrf2 depletion resulted in significantly lower *Aldh2* expression (P < 0.05). Treating the cells with HNE resulted in overexpression of *Gsta4* (eightfold, P < 0.0001) and *Akr1b8* (2.5-fold, P < 0.01) in Co cells, whereas it did not affect *Aldh2* expression. On the other hand, HNE did not affect the expression of these genes in Co-dN cells. Two-way ANOVA followed by Tukey’s multiple comparison test was used for statistical analysis.

#### HPLC profiling and metabolites identification

We treated both cell lines for 30 min with 40 µM HNE containing a radioactive tracer ([^3^H]-HNE) and analyzed the supernatant with radio-high-performance liquid chromatography (HPLC). There are peaks corresponding to HNE and its metabolites, including isomers of glutathione conjugates (HNE-GSH), a mixture of oxidized metabolites (Ox), the saturated derivative of HNE (sat-HNE), and the original HNE molecule, as reported by Baradat et al.^14^. The results, presented as the percentages of observed peaks, showed that HNE metabolites were more abundant in Co cells (P < 0.05), while the remaining original HNE molecule (+ 32%) and its saturated derivatives (+ 14%) were more abundant in Co-dN cells (Fig. 3d). Two-way ANOVA followed by Tukey’s multiple comparison test was used for statistical analysis.

#### Oxidative stress response associated with GSH

**Figure 4:**
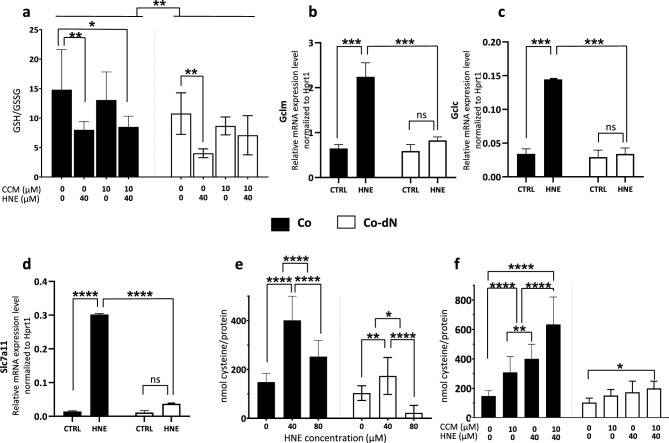
Assessment of GSH-associated oxidative stress response. (**a**) The cellular GSH content is represented by the GSH/GSSG ratio. Where indicated, cells were pretreated with CCM (10 µM for 24 h) and/or treated with HNE (40 µM for 24 h) prior to GSH measurement. (**b**)–(**d**) Relative expression of Nrf2 target genes (*Gclm*, *Gclc*, and *Slc7a11*) in the two cell lines. The detoxification pathway was induced by treating cells with HNE (40 µM for 6 h). Gene expression was normalized to *Hprt1* expression. (**e**) and (**f**) Cystine uptake was assessed after administration of radiolabeled cystine, relative to the total protein content. (**e**) Cells were treated with HNE (40 or 80 µM for 6 h); (**f**) where indicated (CCM and CCM + HNE), cells were pretreated with CCM (10 µM for 24 h) and/or treated with HNE (40 µM for 6 h) prior to radioactive measurement. Black boxes represent Co cells, open boxes Co-dN cells, and error bars indicate the mean ± standard error of the mean (n = 3). Two-way ANOVA followed by Tukey’s multiple comparison test was used for statistical analysis (ns = non-significant, *P < 0.05, **P < 0.01, ***P < 0.001, ****P < 0.0001).

Based on its antioxidant properties, we pretreated cells with curcumin (CCM) for 24 h to induce the cellular antioxidant capacity^15^. Furthermore, CCM is a known inducer of Nrf2^16–18^. The cellular antioxidant capacity is linked to the intracellular GSH content. We determined the GSH/GSSG ratio in both cell lines and after treatments (40 µM HNE and/or 10 µM CCM pretreatment). There was an overall lower GSH/GSSG ratio in Co-dN cells compared with Co cells (Fig. 4a, P < 0.01, − 27% for CTRL, − 50% for 40 µM HNE, − 34% for 10 µM CCM, and − 17% for CCM pretreatment and HNE treatment). These differences reflected greater oxidative stress in Co-dN cells. HNE treatment resulted in a significant decrease in the GSH/GSSG ratio in both cell lines, revealing its oxidative stress effect (− 46% in Co, P < 0.01; − 63% in Co-dN, P < 0.01). Pretreatment with CCM 24 h before HNE treatment did not protect Co or Co-dN cells from HNE-induced oxidative stress (no significant difference between HNE and CCM).

GSH synthesis depends on the regulation of glutamate-cysteine ligase (GCL). Therefore, we analyzed the regulation of the genes that encode the two subunits of this enzyme (*Gclc* and *Gclm*) with RT-qPCR (Fig. 4 b, c). There were no differences between Co and Co-dN cells in the control condition. HNE treatment resulted in a strong induction of both genes in Co cells (P < 0.001). This induction was completely lost in Co-dN cells (P < 0.001).

The balance between cystine uptake and glutamate release, via the glutamate-cystine antiporter xCT, encoded by the *Slc7a11* gene, also regulates cellular GSH levels^19^. We analyzed the regulation of the *Slc7a11* gene by using RT-qPCR (Fig. 4d). There were no differences between Co and Co-dN cells in the control condition, but there was a strong induction by HNE treatment in Co cells (P < 0.0001) but not Co-dN cells.

We then analyzed the cystine uptake using a radioactive tracer ([^14^C]-cystine), treating cells with increasing concentrations of HNE (Fig. 4e) or pretreating cells with CCM (10 µM for 24 h) and then treating or not treating with HNE (40 µM; Fig. 4f). The results, presented as radioactive cystine incorporated into the cells relative to the total cellular protein content, showed that HNE treatment increased cystine uptake in Co and Co-dN cells, but it was significantly lower in Co-dN cells (P < 0.0001). The decreased cystine uptake after treatment with 80 µM HNE could be due to the cytotoxic effect of the molecule (Fig. 4e). Pretreatment with CCM or combination of CCM pretreatment and subsequent HNE treatment induced cystine uptake in Co cells, with higher uptake in the CCM + HNE condition (P < 0.001; Fig. 4f). The effects were virtually abolished in Co-dN cells. Two-way ANOVA followed by Tukey’s multiple comparison test was used for statistical analysis.

### Cell survival under oxidative stress

#### Cytotoxicity

**Figure 5:**
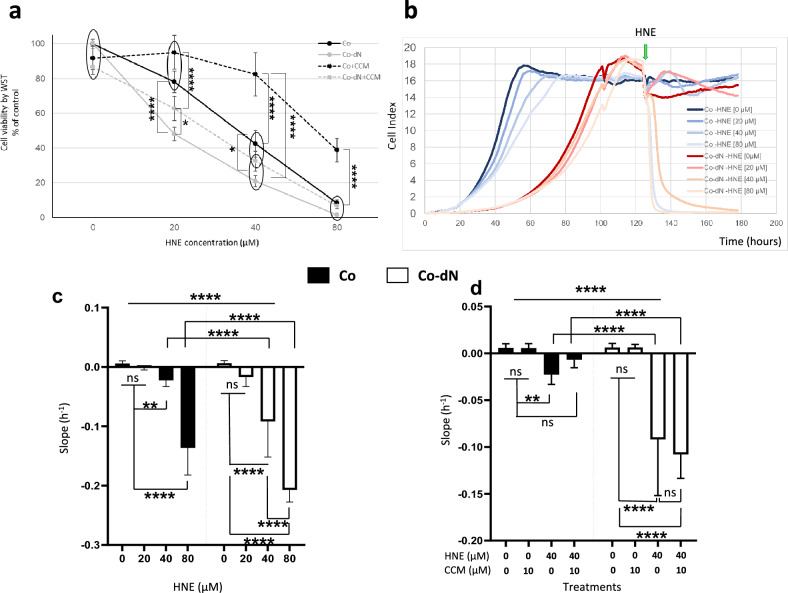
Cell survival in an oxidative context. (**a**) The Co and Co-dN cell lines were treated with increasing concentrations of HNE (0–80 µM), with or without CCM pretreatment (10 µM for 24 h). The results are presented as a percentage of the viability of untreated cells. (**b**) Cell proliferation measured by the xCELLigence technique. Real-time cellular impedance was measured in each well and the signal was analyzed through integrated software (RTCA Analyzer). Average from 5 independent experiments are represented, blue lines are Co cells, red/pink lines are Co-dN cells, in brackets are HNE concentrations. The green arrow represents the initiation time of treatment by HNE. (**c**) and (**d**) Cell proliferation and detachment analyzed using the xCELLigence technique. Increasing concentrations of HNE (0–80 µM) were applied to confluent cells (**c**). Where indicated, cells were pretreated with CCM (10 µM for 24 h) prior to HNE treatment (40 µM for 6 h) (**d**). Black boxes or dots are Co cells, open boxes or dots are Co-dN cells, and error bars indicate the mean ± standard error of the mean (n = 6). Two-way ANOVA followed by Tukey’s multiple comparison test was used for statistical analysis (circles represent nonspecific differences, ns = non-significant, *P < 0.05, **P < 0.01, ****P < 0.0001).

To assess the involvement of Nrf2 in cell viability in an oxidative context, we treated both cell lines with increasing concentrations of HNE (from 20 to 80 µM) during proliferation to represent events that take place deep in the colon crypts. There was a dose-dependent decrease in the survival of both cell lines, with Co-dN cells showing lower viability (20, 40 and 80 µM HNE: 78%, 42%, and 8%, respectively, for Co cells, and 48%, 21%, and 1.5%, respectively, for Co-dN cells; Fig. 5a). Going a step further, we pretreated the cells with CCM (10 µM for 24 h) to induce the Nrf2 transcription pathway. Induction of Nrf2 in Co cells resulted in improved survival (20, 40 and 80 µM HNE: 95%, 82%, and 38%, respectively, for Co cells, and 62%, 32%, and 6.7% for Co-dN cells, respectively). We calculated values equivalent to the IC_50_—that is, the HNE concentration that induced 50% of cell death—to represent the toxic effect of HNE under the different conditions. These HNE concentrations were 35 and 20 µM for Co and Co-dN cells, respectively. Induction of Nrf2 by CCM pretreatment protected the cells, with IC_50_-equivalent concentrations of 70 µM for Co cells and 27 µM for Co-dN cells.

We next used the xCELLigence technique. By this approach, we can monitor cell proliferation, real-time cytotoxicity, and survival of Co and Co-dN cells after HNE treatment and/or CCM pretreatment. This tool is complementary to and more accurate than the cell proliferation test. Both cell lines were treated after reaching confluence with an increasing concentration of HNE (from 20 to 80 µM) (Fig. 5b). Positives values of the slope represent growing cells, while a negative slope value reflects a decrease in the number of cells. The decrease in the number of cells was proportional to the HNE concentration in Co cells (P < 0.02 for 40 µM HNE and P < 0.0001 for 80 µM HNE; Fig. 5a). Co-dN cells were more sensitive to the toxic effects of HNE (P < 0.0001 for 40 and 80 µM HNE), with no significant difference between the two concentrations. The difference between the two cell types was highly significant (P < 0.0001) (Fig. 5c). Taken together, CCM alone had no significant effect on cell growth, but it did reduce cellular toxicity induced by 40 µM HNE in Co cells, with no significant difference between CCM + HNE and CCM alone, but a significant difference compared with HNE 40 µM (P < 0.006) (Fig. 5d). CCM did not prevent toxicity in Co-dN cells. Two-way ANOVA followed by Tukey’s multiple comparison test was used for statistical analysis.

#### Genotoxicity

**Figure 6:**
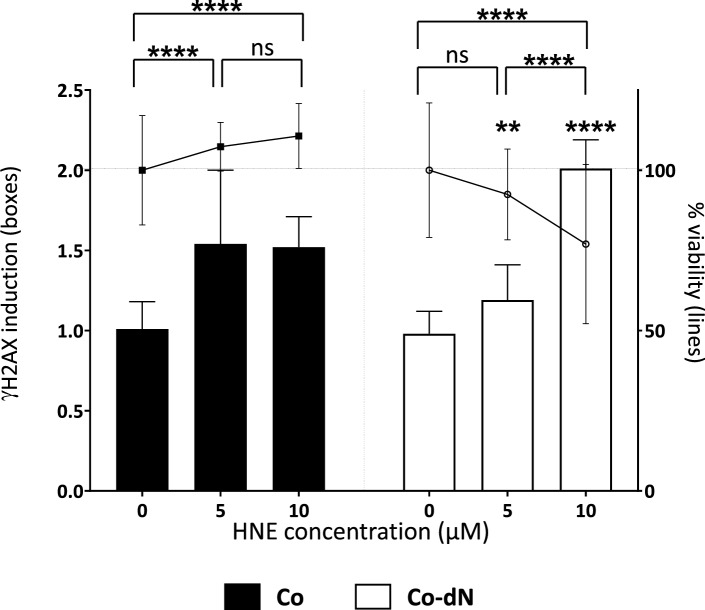
The genotoxic effect of HNE was determined by measuring γH2AX induction. γH2AX induction is represent by bars (left Y axis). Cell viability was assessed in parallel (right Y axis), represented by lines. Cells were treated with increasing concentrations of HNE (0–10 µM). Black boxes represent Co cells, open boxes represent Co-dN cells, and error bars indicate the mean ± standard error of the mean (n = 3). Two-way ANOVA followed by Tukey’s multiple comparison test was used for statistical analysis (ns = non-significant, **P < 0.01, ****P < 0.0001).

We determined the genotoxic effect of HNE on both cell lines by measuring the induction of γH2AX. Cells were treated with increasing concentrations of HNE, without reaching cytotoxic doses (i.e., 5 and 10 µM), and the percentage of relative cell count (%RCC) was determined. The genotoxic effect of HNE was greater in Co-dN cells. The induction of γH2AX was 1.5-fold in Co cells (both HNE concentrations) and 1.2-fold at 5 µM HNE and twofold at 10 µM HNE in Co-dN cells (Fig. 6).

## Discussion

The main objective of this work was to generate a suitable cellular model, based on a non-tumoral murine cell line, to explore the role of Nrf2 in the cellular toxicity induced by food contaminants or neo-formed compounds (such as lipoperoxidation products) from digestion. The development of a cellular model free of cancer-related mutations is essential for studying the responses of normal cells to xenobiotic stimuli, including genotoxic agents, and for identifying potential cancer-initiating molecules. As Nrf2 is the main regulator of a panel of antioxidant and cell protection genes, mitigating cellular damage due to oxidative stress, a Nrf2-depleted cell line could allow researchers to assess the implication of Nrf2 in the cellular response to oxidative stress. To our knowledge, we are the first group to generate Nrf2-deficient normal murine epithelial colonic cells. Following CRISPR/Cas9-mediated invalidation of Nrf2, we used HNE as a representative toxic lipid peroxidation product, reflecting the heme-induced cytotoxic and genotoxic alkenals produced in the colon during digestion, to highlight the importance of Nrf2 in cellular protection against toxic injuries^9^. In an attempt to identify the mechanisms involved in these processes, we focused on the GSH pathway, HNE metabolism, target gene modulation, and the cell proliferation capacity. We also used CCM, a well-known inducer of Nrf2^16,18^, to confirm our results.

The Co cell line used in these experiments was derived from the Apc^+/+^ clones described by Forest et al.^8^ and characterized by Plaisancié et al.^20^. To ensure the non-transformed status of our cell line, we performed WGS, focusing specifically on cancer-related genes. Despite a relatively low coverage rate for our sequences, we carried out an in-depth analysis on cancer-related genes to ensure that coverage was sufficient for our analyses. We used the snpEff classification of variants to identify mutations in the tested genes. We compared the Co cell sequences to the C57Bl/6J mouse genome, focusing on genes from different pathways involved in cancer hallmarks. None of these genes were mutated in our cell line. We paid particular attention to the WNT pathway as *Apc* mutations are involved in a majority of human colorectal cancer cases^21^. Moreover, the WNT pathway has been shown to activate Nrf2^13^. We also tested tumor suppressor genes such as Tp53 and proto-oncogenes such as those in the phosphoinositide 3-kinase (PI_3_K) pathway. Among all of the tested genes, listed in the Tables S3 and S4, only 3 differed from the wild-type. The identified variants associated with Notch4 and Tp53 are non-disruptive variants and the variants with disruptive effect on protein (high impact) were associated with Nrf2, confirming that our cell line is cancer-free^22^.

The CRISPR/Cas9 technique has rapidly become a reference method for generating mutations in specific genes, leading to gene silencing^23^. Comparison in isogenic cell lines of the disruption of a specific gene is particularly interesting for studying specific pathways. After selection, we obtained a Nrf2-depleted clone, which we called Co-dN. Our data confirmed that Nrf2 protein knockdown was successful in these cells. Although Nrf2 mRNA was still partially detectable (Fig. 1a), the protein was not detectable under our conditions, even after HNE stimulation. The Co and Co-dN cell lines are isogenic, and we confirmed the absence of genetic differences between them by performing WGS. Comparing the difference in high impact variants in Co-dN and Co cells, only two genes were impacted, namely Nrf2 and Cdk6. Thus, we confirmed that the CRISPR/Cas9 technique was able to generate a Nrf2-depleted cell line, with no off-target mutations (Fig. 2; Table 1). Only a few cases of functional knockout of the Nrf2 gene by CRISPR/Cas9 have been reported—in human lung cancer cells (A549), human embryonic stem cells^24,25^, and, recently, in the human colorectal cancer cell line DLD1^26^, where isogenic wild-type cells, CRISPR/Cas9 generated Nrf2-KO cells and gain-of-function Nrf2 cells were compared. HTC 116 cells, a human colon cancer cell line, have also been used to identify p53 protein activity in drug-induced colon cancer cell death^27^. Rayner and colleagues^28^ reported that the CRISPR/Cas9 technique causes chromosomal instabilities and rearrangements in cancer cell lines.

We had previously shown that heme iron, derived from red meat consumption, can promote colorectal cancer, probably due to its strong ability to catalyze the peroxidation of dietary polyunsaturated fatty acids (PUFAs), generating cytotoxic and genotoxic alkenals in the colon^29^. These molecules have a longer half-life than ROS and are considered second messengers of oxidative stress^2,3^. Among the reactive aldehydes produced by lipid peroxidation, malondialdehyde (MDA) and HNE are the major products. HNE, the end-product of n-6 PUFA peroxidation, is considered one of the most bioactive aldehydes^2,30^ and the most cytotoxic to colon cells^14,31,32^. High levels of HNE in the cytosol can activate Nrf2 via its adduction onto Keap1 cysteines, inducing conformational changes, followed by Nrf2 release and translocation to the nucleus, where its binds to the antioxidant-responsive element (ARE) present in the promoter of several genes^3,31^. Nrf2 has been shown to regulate the expression of antioxidant enzymes, resulting in an adaptive response to alkenal-related injury^3,33,34^. Furthermore, HNE has been shown to activate Nrf2 in a variety of cells, including colon cancer cells^2,26,35–38^. In the present study, we used HNE to decipher the molecular mechanisms involving Nrf2 in cellular protection against cytotoxic molecules resulting from heme-induced lipoperoxidation.

We assessed three of the main HNE detoxification pathways in epithelial cells by analyzing the enzymes Gsta4, Aldh2 and Akr1b8^31^. Gsta4 is the glutathione-*S*-transferase specific for HNE conjugation to GSH and the main cellular HNE detoxification pathway. It is highly expressed in the neoplastic epithelia of invasive carcinomas and is activated by HNE and Nrf2^39^. Akr1b8 is responsible for the reduction of reactive aldehydes to their less toxic alcoholic forms^40^. Aldh2 participates in the oxidation of the carbonyl group of HNE leading to the formation of 4-hydroxy-2-nonenoic acid (HNA)^41^. The results showed the involvement of Nrf2 in the HNE detoxification process involving Gsta4 and Akr1b8, but not Aldh2. To further investigate the role of Nrf2 in detoxification processes, we treated cells with radiolabeled HNE. In Co-dN cells, the original HNE molecule and its saturated derivatives tended to be more abundant, while HNE-GSH and oxidized metabolites were less present, demonstrating that Nrf2 is the cornerstone of the HNE detoxification process.

GSH is one of the most important components of the cellular antioxidant defense against many reactive compounds. It involves the cystine/glutamate antiporter xCT encoded by *Slc7a11*. Imported cystine is reduced by GCL, via the catalytic and modulatory subunits (Gclc/Gclm), and glutathione synthetase (Gss) to generate GSH^34^. In addition, HNE is detoxified via conjugation with cysteine to form a non-toxic HNE-cysteine conjugate^14,41^. Tonelli et al.^42^ reported that Nrf2 tightly regulates GSH levels and directly controls the expression of the GCL complex (Gclc/Gclm), playing a fundamental role in maintaining cellular redox homeostasis, in addition to regulating GSH synthesis. We measured the GSH/GSSG ratio; *Gclm*, *Gclc*, and *Slc7a11* gene expression; and cystine uptake in Co and Co-dN cells after HNE treatment and with or without CCM pretreatment. Taken together, the results presented in Figs. 3 and 4 demonstrate that the detoxification process involved in the response of mouse colonic cells to HNE-induced injury involves the GSH pathway, and depletion of Nrf2 results in reduced cell protection against toxic injuries. HNE can induce genes involved in its detoxification process (*Slc7a11* and *Gsta4*)^25,34,41,42^, and Nrf2 activation may be linked to this process, as it has been suggested that HNE modifies the cysteine residues of Keap1, the negative regulator of Nrf2^26,36,41^. Okazaki et al.^43^ reported that in cancer cells, activation of Nrf2 induces transcriptional activation of *Slc7a11*, *Gclc*, and *Gclm* in response to oxidative and electrophilic stresses, such as HNE. For these authors, cancer cells are addicted to Nrf2 and adopt a highly specialized metabolism favoring GSH synthesis, and therefore have powerful antioxidant detoxification capabilities. These changes in cellular metabolism, combined with activation of the Nrf2 pathway to promote a highly efficient detoxification process in cancer cells, could be linked to a greater resistance to anticancer drugs.

We demonstrated the importance of Nrf2 in cell survival after HNE injury, as increasing concentrations of HNE resulted in the decreased survival of Co and Co-dN cells, with Co-dN cells showing greater sensitivity. Induction of Nrf2 by CCM protected Co cells, as the IC_50_-equivalent concentration doubled (from 35 to 70 µM). However, CCM had virtually no effect on Co-dN cells. It is important to note that we cannot rule out the possibility that CCM may act on other cellular pathways.

Nrf2 overexpression is known to induce cell proliferation^24,44,45^. We used the xCELLigence technique to study the rate of cell proliferation as well as cell viability. Nrf2 depletion led to slower cell growth, confirming the importance of Nrf2 for the proliferation rate in our model. Furthermore, this technique confirmed the toxic effects of HNE (Fig. 5b, c). Pretreatment of cells with CCM prior to HNE treatment protected cells from HNE-induced damage, and had no effect on Co-dN cells (Fig. 5c). These results confirm those obtained in the viability tests (Fig. 5a).

We also showed that Nrf2 could modulate the genotoxic activities of HNE. The genotoxic effects of HNE have already been reported^30,31,46^. In our cells subjected to non-cytotoxic concentrations of HNE, there was greater genotoxic damage in Co-dN cells. It could be of great interest to investigate whether HNE, through its Nrf2-activating effects, could be considered a cancer-initiating agent.

We used CCM, a known inducer of Nrf2^16,18^, to confirm the involvement of Nrf2 in cell protection against oxidative stress. This phytochemical has been shown to stabilize Nrf2 protein in mouse epidermal cells^18^. This results in activation of Nrf2 and induction of cytoprotective targets genes. Thus, CCM could act as an antioxidant and anti-inflammatory compound, counteracting ROS formation^15^. Considering the “double-edged sword” action of Nrf2^47^, it is interesting to evaluate the effects of Nrf2 stimulation in non-cancerous cells. In Co cells, CCM exerted protective effects, notably by protecting cells from HNE-induced damage leading to cell death. Because of its antitumor and Nrf2-inducting activities, this natural compound has the potential to be used to prevent colorectal cancer^15^.

Certain limitations need to be considered when interpreting our results. The strategy for creating a conditionally immortalized murine colon cell line involved introducing the SV40 large T antigen as the immortalization gene, which carries a temperature-sensitive mutation. At the permissive temperature of 33 °C, the conformation of the SV40 large T protein enables it to bind to the p53 protein, inhibiting its role in senescence and thus facilitating cell immortalization. Therefore, cells need to be cultured at 33 °C, which is not the physiological temperature. When cells are cultured at 37 °C (the non-permissive temperature), the SV40 large T protein no longer binds to p53, and the cells can partially differentiate into colonocytes. Furthermore, the HNE concentrations we used are not physiological concentrations found in the colon lumen. However, we used this molecule as a representative of all the toxic alkenals that might be present in the colon lumen. The CRISPR/Cas9 technique led to an incomplete deletion of Nrf2 due to the random insertion/deletion in the genomic sequence. Nevertheless, the Co-dN cells with partial deletion of Nrf2 could be considered a more realistic model given the evidence for the Nrf2 decline with aging.

The cellular model of the non-cancerous Co cell line was enhanced by the generation of an isogenic cell line with Nrf2 depletion, enabling us to highlight the importance of this transcription factor in the cellular response to oxidative stress-induced molecules, and in particular the involvement of GSH in the detoxication of oxidative stress. Oxidative stress is a major feature of aging, in which the ability to respond to oxidative stress is reduced. Interestingly, during aging, basal Nrf2 protein levels and activity are reduced, as is the expression of genes such as those of the GCL family and Hmox-1^48^. Decreased Nrf2 expression and transcriptional activity may contribute to the loss of homeostasis and affect function that correlate with aging^49–51^. Thus, this dual-cell model represents an original tool for studying the role of Nrf2 in the response to oxidative stress induced by food-derived xenobiotics, luminal peroxidation, and ROS^52^, and could be used to explore metabolic perturbations that emerge during aging.

## Materials and methods

### HNE synthesis

HNE was synthesized as previously described^53^. Briefly, the Grignard reaction between fumaraldehyde monoacetal and 1-pentylmagnesium bromide afforded HNE-dimethylacetal. HNE was obtained by acid hydrolysis.

### Cell culture conditions

Murine colon epithelial cells were used as described in Surya et al.^32^. The Co cell line used in these experiments was issued from the Apc^+/+^ clones described in Forest et al.^8^. This transformed cell line firstly generated by Pierre^8^ is present in the lab since then. Cell lines were cultured at permissive temperature of 33 °C in Dulbecco-modified essential medium (DMEM) supplemented with 10% fetal calf serum, 1% penicillin/streptomycin, 2% glutamine, 10 U/ml interferon γ and 10 U/ml epidermal growth factor (EGF) (Merck, France). Excepted when indicated, most experiments were performed at non-permissive temperature of 37 °C without INFγ and EGF to inhibit the SV40 transgene and to reach a differentiated state. HNE treatments were performed in serum-free DMEM containing 2% glutamine for 24 or 6 h as indicated in the experiments. Pretreatment with curcumin (CCM, 10 µM) was performed the day before HNE treatment at 37 °C for 24 h.

### Whole genome sequencing

Genomic DNA for whole-genome sequencing (WGS) was extracted from murine cells using the GenElute Mammalian Genomic DNA Miniprep Kit as described by the manufacturers (Sigma, G1N10). DNAseq was performed at the GeT-PlaGe core facility, INRAe Toulouse. DNA-seq libraries have been prepared according to Illumina’s protocols using the Illumina TruSeq Nano DNA HT Library Prep Kit. Briefly, DNA was fragmented by sonication, size selection was performed using SPB beads, and adaptors were ligated to be sequenced. Library quality was assessed using an Advanced Analytical Fragment Analyzer and libraries were quantified by qPCR using the Kapa Library Quantification Kit. DNA-seq experiments have been performed on an Illumina NovaSeq6000 using a paired-end read length of 2 × 150 pb with the Illumina NovaSeq6000 Reagent Kits. The raw sequences were deposited at SRA under accession PRJNA800346.

### Bioinformatics analysis

FastQC^54^ was used to check the quality and no relevant contamination hit was found after the alignment against E. coli, Yeast and PhiX. Reads mapping and variants calling were performed using Nextflow v20.10.0 and Sarek pipeline v2.6.1^55^. Genome Analysis Toolkit (GATK) best practices were followed as implemented in the Sarek pipeline. The following steps were performed: aligning the reads with BWA v0.7.17-r1188^56^ against the mouse genome assembly C57BL_6NJ_v1 (GCA_001632555.1), marking duplicate reads (MarkDuplicates), base quality recalibration (BQSR) and calling germline small variants (HaplotypeCaller in GVCF mode) with GATK v4.1.7.0^57^ on merged bam by sample. Finally, quality control is performed with MultiQC v1.8^58^. Out of the nextflow pipeline the variants were annotated with VEP(5) version 103.1 with assembly C57BL_6NJ_v1. Variant with at list quality of 30 and depth of 5 were kept to further analyses.

### Nrf2 invalidation

The CRISPR/Cas9 technique used to generate the Nrf2-depleted cell line used the 3 “all-in-one plasmids” containing 3 pre-designed gRNAs (Sigma, Merck Company) that target 3 different sequences at exon 2 of the murine Nrf2 mRNA gene, transcript variant 1, NM_010902.4 (Supplementary Fig S1a). These gRNAs coupled with promoters and the GFP (Green Fluorescent Protein) were inserted into vector plasmids (pCMV-cas9-GFP). These plasmids where amplified and purified (Column Plasmid DNA Mini-preps Kit, BioBasic). Cells were transfected 48 h post plating (30 000 cells/cm2) using X-treme GENE HP DNA Transfection Reagent (Roche) with a ratio of 2 µl XtremeHP/2 µg plasmid DNA. Cells were treated with the three different gRNAs. Transformed cells were analyzed and separated into three distinct GFP positive populations (weak, medium and high) using cell sorting flow cytometry (FACSARIA-II, BD Biosciences, CPTP Toulouse). Only the populations that integrated the largest amount of plasmid (High GFP) were plated at one cell per well of 96-well culture plates rate. DNA mutations in Nrf2 target gene were sought by PCR using primers specific to each gRNA (Supplementary Fig. S1).

### Western blot

Nrf2 expression in whole cell protein extracts was determined by Western blot. Two sets of cells (Co and Co-dN) were treated, or not, by HNE (40 µM for 6 h) before protein extraction. Protein concentration was determined using BCA protein Assay kit (Pierce, Rockford, II, USA). Proteins were separated using an SDS-PAGE gradient pre-cast gels 4–12%, and transferred onto a nitrocellulose membrane 0.2 µM (Trans-blot turbo mini 0.2 µM nitrocellulose Transfer pack) using Trans-Blot® Turbo™ (7 min, 25v, 1.3 A) (all from Bio-Rad). Detection of the primary antibody (Nrf2 rabbit polyclonal IgG, Genetex Biotech, 1:500) and vinculin (rabbit IgG, Proteintech Europe, 1:5000) was performed with the WesternBreeze system (WesternBreeze™ Chemiluminescent Kit, anti-rabbit for Nrf2 and vinculin, Thermofisher). Protein bands were visualized using Chemidoc with the associated software (Bio-Rad, ImageLab 5.2.1 software) and the intensity of each bands were evaluated. Full size, images and band intensity are shown in supplementary Fig. S2 and Table S2.

### Gene expression

Total cellular RNA was extracted with Tri reagent (Molecular Research Center). Total RNA samples (1 µg) were then reverse-transcribed with the iScript™ Reverse Transcription Supermix (Bio-Rad) for real-time quantitative polymerase chain reaction (qPCR) analyses. The primers for Sybr Green assays are presented in Supplementary Table S1. Amplifications were performed on a ViiA 7 Real-Time PCR System (Applied Biosystems). The qPCR data were normalized to the level of Hypoxanthine Phosphoribosyl transferase − 1 (Hprt1) messenger RNA and analyzed by LinRegPCR v.11 software.

### GSH/GSSG assay

The GSH/GSSG ratio was evaluated with the GSH/GSSG™ Assay (Promega, France) based in a luminescence detection of GSH-dependent conversion of a GSH probe, Luciferin-NT, to luciferin by a glutathione S-transferase enzyme coupled to a firefly luciferase reaction. Cells were seeded into a 96-well plate. After treatment, total glutathione and oxidized glutathione were measured according to the manufacturer’s instructions. Results are expressed as GSH/GSSG ratio.

### Cystine uptake assay

L-[^14^C]-Cystine (PerkinElmer, 100 mCi/mmol) uptake was measured according to Giraudi et al. with some modifications^59^. Cells were seeded in a 12-well plate pretreated with CCM and incubated for 6 h with HNE as previously described. Cells were then rinsed with warmed uptake buffer. Cystine uptake was started by incubating the cells in uptake buffer containing L-[^14^C]-Cystine 0.1 μCi at room temperature for 10 min, and stopped by rinsing with ice-cold unlabeled uptake buffer. Cells were then lysed by adding NaOH 0.1 N. Lysate was mixed with a scintillation cocktail (UltimaGold, PerkinElmer), protein quantification was done with the BCA protein Assay kit (Pierce, Rockford, II, USA) and radioactivity was determined using a scintillation counter (Hewlett Packard). Results were expressed as nmol of incorporated cystine reported to total protein content.

### Cellular viability

Cell viability assay was performed by using cell proliferation reagent WST-1 (Roche Life Science) according to manufacturer’s protocol. Briefly, cells were seeded and grown in 96-well plates at 33 °C until they reach 50% confluence, then transferred at 37 °C in the non-permissive medium with 10 µM curcumin when indicated (CCM, HC) for 24 h. HNE was then added (0–20–40 and 80 µM) for the next 24 h in a serum-free medium at 37 °C. Cells were incubated in WST-1 reagent for 1 h in the dark. The absorbance was evaluated at 440 nm using a Tecan apparatus. The results from 6 independent experiments were expressed as percentage of non-treated control cells.

### Cellular genotoxicity

Genotoxic effects of increasing concentrations of HNE was measured by γH2AX in-cell Western assay according to Khoury et al.^60^. Cells were seeded into 96-well plates at 15 × 10^3^ cells per well in permissive conditions. After reached subconfluence, cells were transferred to 37 °C and treated with HNE (0, 5, 10 µM) for 24 h. The results are expressed as γH2AX fold induction. To consider the cytotoxicity of HNE, the percentage of relative cell counts, measured as DNA content had been evaluated and the viability curves were added to the graphs.

### Real-time cellular impedance

The real-time cell impedance analyzer (xCELLigence system) was used according to the manufacturers’ instructions (Roche Applied Science, Mannheim, Germany, and ACEA Biosciences, San Diego, CA, USA). Each cell line was added to the 16-well E-plates at exactly 10 000 cells per well. After 96 h at permissive conditions, when cells had reached confluence, the medium was changed to non-permissive medium and temperature, without serum and different HNE concentrations (20; 40; 80 µM). When indicated, cells were pretreated with curcumin (CCM, 10 µM, complete medium, permissive conditions) 24 h before HNE treatments. Real-time cellular impedance was measured in each well (cell index values) and the signal was analyzed through integrated software (RTCA Analyzer). The results are indicated as the slope (h − 1), with positive values representing proliferating cells (increasing cell number), and negatives values represent detachment of cells (decreasing cell number). Results are from 5 independent experiments.

HNE metabolism was studied using radiolabeled HNE synthesized as previously described^61^ (specific activity 222 GBq/mol). Cells were treated by 40 µM [^3^H]-HNE for 30 min, then cellular supernatants were analyzed by radio-HPLC as described before^14^, except for the HPLC gradient used that has been modified to improve peak separation: both mobile phases contained water/acetonitrile/acetic acid with phase A (97.5/2.5/0.1) and B (40/60/0.1); the elution gradient was 0 min 18% B, 2–10 min 26% B, 23–37 min 41% B, 42 min 65% B, 47 min 100% B, 60 min 100% B, 65 min 18% B.

### Statistical analysis

All data were expressed as mean ± standard error of the mean (SEM) of three (or more) independent experiments. Statistical significance was determined by two-ways analysis of variance (ANOVA), followed by Tukey’s multiple comparison test when needed, except for Fig. 1a and 1c, where a t-test had been performed, using GraphPad Prism 9 software. Statistical significance was indicated by *P < 0.05, **P < 0.01, ***P < 0.005 and ****P < 0.001.

### Supplementary Information

Supplementary Information.

## Data Availability

All data associated with this study are present in the paper or the supplementary data, and are available from the corresponding author upon reasonable request. The datasets generated (raw sequences) and analyzed during the current study are available in the NCBI/SRA repository under accession PRJNA800346.
