# Supplementary material for: A dual model of normal vs isogenic Nrf2-depleted murine epithelial cells to explore oxidative stress involvement
Source: Sci Rep. 2024 May 13;14:10905. doi: 10.1038/s41598-024-60938-2 (PMC11091076; doi:10.1038/s41598-024-60938-2)
Supplement: Supplementary file 1 — Supplementary Information. [file 41598_2024_60938_MOESM1_ESM.pdf]

	Forward	Reverse
**Hprt1**	TCATAACCTGGTTCATCATCGC	GCCGACCCGCAGTCC
**Nrf2 (Nfe2l2)**	ATTGCCACCGCCAGGACTA	TGCCTCCAAAGGATGTCAATCAAA
**Hmox1**	CAACCCCACCAAGTTCAAACA	AGGCGGTCTTAGCCTCTTCTG
**Gsta4**	GAAGTTCTAGTGACAGCGTGCTTTA	TGTAGCTGCTGCTGTGATTGG
**Akr1b8**	GCCGCCAAGCACAAGAAAA	CCTCTGGATGTGGAACCGAAT
**Aldh2**	GCCGCAGACCGTGGTTACT	CGATGGTCATGCCATCTTTG
**Glcm**	CTGCTAAACTGTTCATTGTAGG	CTATTGGGTTTTACCTGTG
**Gclc**	GTCTTCAGGTGACATTCCAAGC	TGTTCTTCAGGGGCTCCAGTC
**xCT (Slc7a11)**	CCTGGCATTTGGACGCTACAT	TCAGAATTGCTGTGAGCTTGCA

Supplementary Table S1: Primer sequences for RTqPCR assays.

a

Target ID	Left sequence	Right sequence	Amplicon size (nt)
MM0000304356	TGATTGACATCCTTTGGAGGC	TGTGTCCTGATTATCAAGAAGCG	398
MM0000304357	TTGATTGACATCCTTTGGAGGC	CCTCCCTGTGATCTGTGTCC	412
MM0000304358	GACTTTAGTCAGCGACAGAAGG	CCTCCCTGTGATCTGTGTCC	355

b

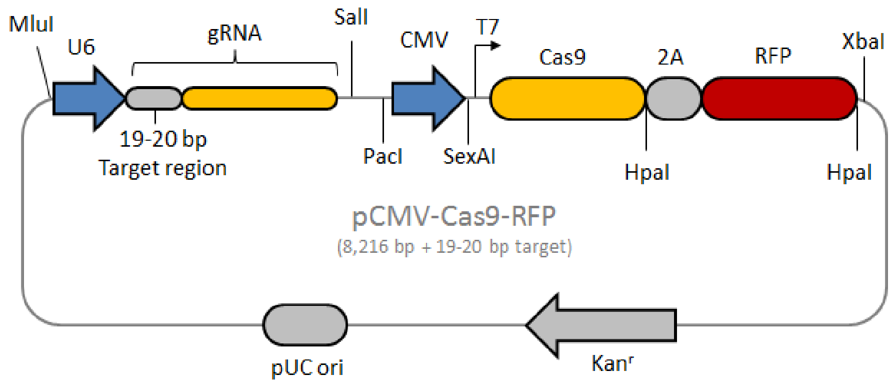

c

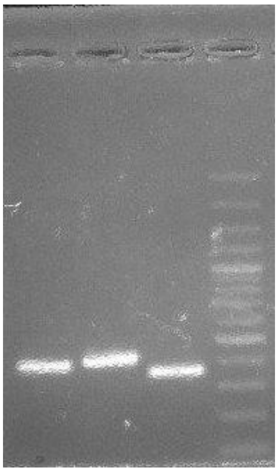

304356 304357 304358

Supplementary Figure S1: **CRISPR /Cas9 information,** from Sigma-aldrich.com. **a.** Sequence of the 3 guide RNAs (gRNA) used (see location on Fig1a) **b.** All-in-one plasmid (scheme from Sigma Aldrich); **c.** Separation and isolation of the different fragments on agarose gel after PCR.

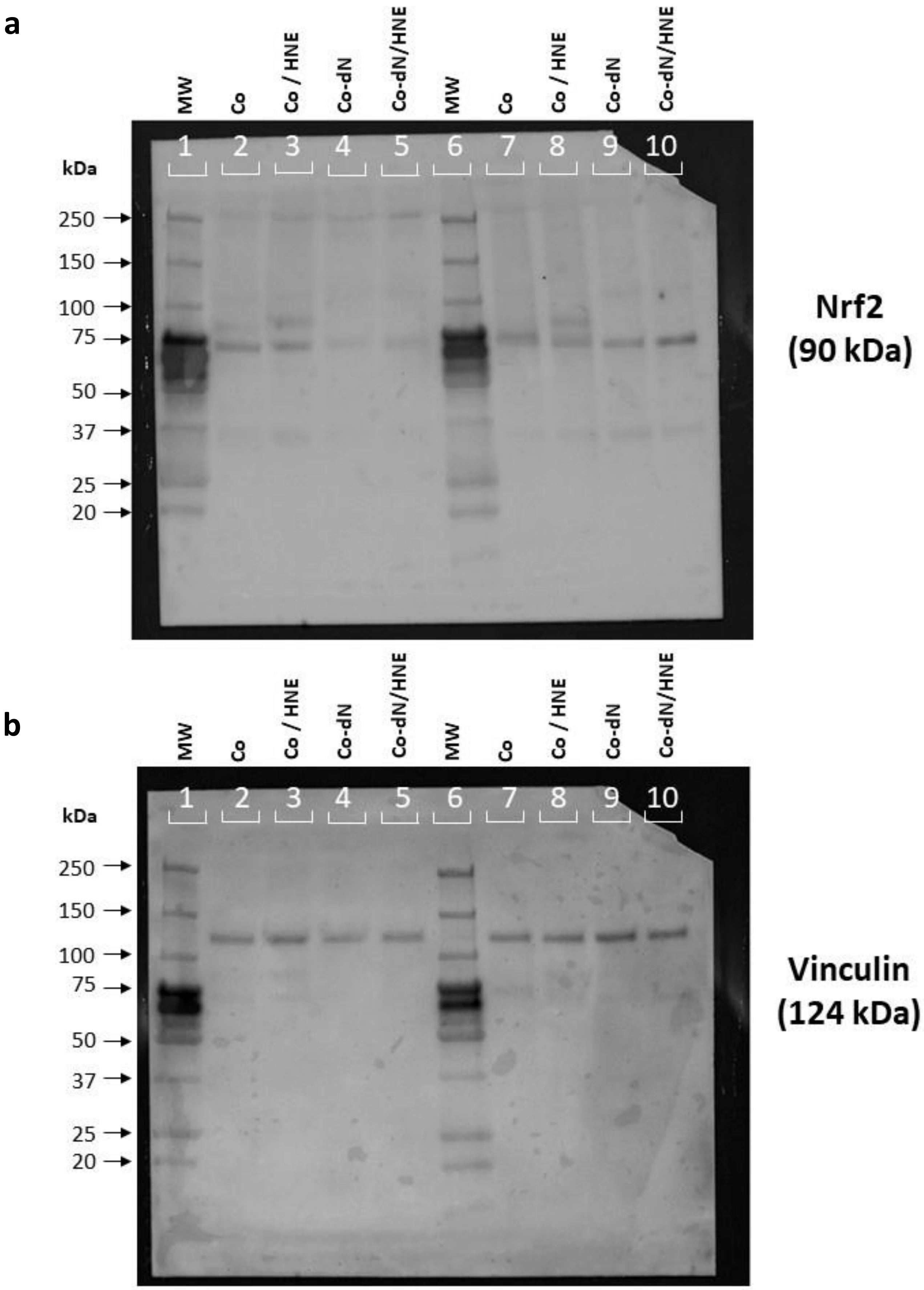
a
MW
Co
Co / HNE
Co-dN
Co-dN/HNE
MW
Co
Co / HNE
Co-dN
Co-dN/HNE
1 2 3 4 5 6 7 8 9 10
kDa
250
150
100
75
50
37
25
20
Nrf2
(90 kDa)
b
MW
Co
Co / HNE
Co-dN
Co-dN/HNE
MW
Co
Co / HNE
Co-dN
Co-dN/HNE
1 2 3 4 5 6 7 8 9 10
kDa
250
150
100
75
50
37
25
20
Vinculin
(124 kDa)

Supplementary Figure S2: Full-length Western blot analysis of protein expression in Co and Co-dN cell lines. Two sets of cells were treated without (Co; Co-dN) or with (Co / HNE; Co-dN / HNE) HNE (40 µM, 6h). the first set was loaded on lines 2 to 5, the second set on lines 7 to 10. **a**. Antibody against Nrf2 (90 kDa), **b.** Vinculin (120 kDa) was used as internal control.

cells	Lines 2 to 5	%	Lines 7 to 10	%
**Co**	8.19	100%	7.62	100%
**Co /HNE**	19.33	236%	15.15	199%
**Co-dN**	0	0	0	0
**Co-dN /HNE**	0	0	0	0

Supplementary Table S2. Nrf2/Vinculin ratio. Protein bands were visualized using Chemidoc with the associated software (Bio-Rad, ImageLab 5.2.1 software) and the intensity of each bands were evaluated (relative values). The Nrf2/Vinculin ratio was calculated and the percentage of HNE induction is reported.

a : Raw data

Sample Name	Raw reads	% Duplicates	Reads Aligned	≥10X	Mean cov	Vars	SNP	Indel
**Co**	481,788,378	29.6%	479,112,014	45.03	17.39	1,354,919	993,532	362,827
**Co-dN**	552,931,396	17.2%	550,445,478	81.51	24.04	1,562,679	1,113,680	450,925

b : Variant detection

**Pathways**	**Genes tested**	**Transcript Length**	Average depth (*Mosdeth*)		Feature count on exons		Gene filter	Moderate	High	Gene filter	Moderate	High
			**Co**	**Co-dN**	**Co**	**Co-dN**	**Co**			**Co-dN**		
**WNT**	APC	15 753	14.27	18.80	1 710	2 781	9			11		
	CTNNb1 / Beta catenin	5 099	50.23	20.04	736	830	5	1		4	1	
	GSK3 beta	9 821	11.49	19.79	3 248	1 856	10			38		
	Axin 1	3 915	28.44	27.70	1 662	1 179	14			9		
	Axin 2	5 506	36.50	23.40	2 768	1 558	7			3		
**NOTCH**	NOTCH 1	14 232	38.73	19.05	7 990	3 429	12	1	1	11		
	NOTCH 2	10 777	24.76	33.55	4 501	4 277	20			17		
	NOTCH 3	8 044	64.99	28.36	6 399	2 865	10	1		8		
	NOTCH 4	8 080	35.11	18.20	5 541	2 801	43			77	1	
**myc**	c-myc	2 767	35.26	20.85	1 155	660	1			2		
**TGF beta**	SMAD 2	2 037	11.51	23.34	104	619	4			7		
	SMAD 3	5 542	64.97	22.56	883	1 273	11			18		
	SMAD 4	3 741	21.34	25.51	631	1 053	12	1		10		
	BMP4	2 159	75.70	14.65	1 411	410	3			3		
**Hippo**	Yap1	9 257	13.06	21.19	1 978	1 645	38			17		
**PI3K**	Akt1	3 948	14.27	19.27	2 955	608				3		
	BRCA1	8 533	13.15	27.09	786	2 529	9			23		
**P53**	TRP 53	4 056	29.77	26.42	1 069	931	6			5	1	
**Cell cycle**	Cdk2	2 432	71.45	23.68	1 385	519	2			1		
	Cdk4	3 249	13.52	18.04	1 978	647	1			1		
	Cdk6	3 151	33.03	44.15	1 132	1 401	2 678	12	3	2954	15	4
	E2f1	3 150	87.34	50.00	2 670	1 402	13			5		
	E2f3	4 794	38.12	11.71	790	888	8			13		
	cdkn2a	1 172	48.88	16.97	893	276	9			5		
**others**	B raf	13 863	12.48	26.81	1 755	3 914	20			14		
	K ras	5 347	25.54	21.88	1 035	1 249	1			2		
	PTEN	10 632	16.14	23.24	1 060	2 247	7			12		
	COX-2	4 580	12.54	21.28	672	1 146				1		
	Fas	1 801	14.84	27.38	169	636	10			7		
	Bcl2	9 129	7.14	23.95	2 195	1 857	13			16		
**NRF2**	Nrf1	5 350	83.79	28.40	3 783	1 425	2	1		1		
	Nrf2	3 796	20.67	17.40	702	716	4			5	1	2
	Nrf3	2 973	43.92	27.78	1 663	986	8			9		
	Keap1	4 664	24.99	14.67	2 978	969	7	1		5	1	

Supplementary Table S3: **Sequence comparison analyses**. **a.** Raw data. **b.** Variant detection in a panel of genes from the main colorectal cancer–related pathways.

	Co		Co-dN	
**Gene symbol**	***Moderate***	***High***	***Moderate***	***High***
Cdk6	12	3	15	4
Ctnnb1	1		1	
Keap1	1		1	
Gm16505 *	***7***	***5***	***16***	***6***
Nfe2l1	1			
Nfe2l2§			1	2
Notch1$^{\#}$	1	1		
Notch3$^{\#}$	1			
Notch4§			1	
Smad4$^{\#}$	1			
Trp53§			1	

Supplementary Table S4: Detected variants in Co and Co-dN cells. * predicted gene Gm6505, $^{\#}$ Co specific variants, § Co-dN specific variants.
